# Hydrovoltaics brings a new solution in alternative energy: an interview with Prof. Wanlin Guo

**DOI:** 10.1093/nsr/nwae096

**Published:** 2024-03-23

**Authors:** He Zhu

## Abstract

The Water Hub Project at the Three Gorges, also known as the ‘Sanxia Project’, now provides more than 1 billion kilowatt-hours of electricity daily to 10 provinces in eastern China. Facing rising energy demands and climate change, despite being the largest hydroelectric power station in the world, this project may still not live up to the vision of the inventor of hydroelectric power, Nikola Tesla, when he said: ‘Electric power is everywhere present in unlimited quantities and can drive the world's machinery without the need of common fuels.’ Hydrovoltaic technology, invented by Prof. Wanlin Guo of Nanjing University of Aeronautics and Astronautics (NUAA), aims to generate electricity through processes such as evaporation and the motion of water droplets on synthetic nanomaterials. *National Science Review* recently invited Prof. Guo for an in-depth interview to discuss this exciting new technology and how it may represent the next great opportunity to transition from fossil fuels to renewable energy sources.

***NSR***: Thank you for making time for this interview. How did the hydrovoltaic research emerge from your previous work on fatigue and cracks in aviation materials at NUAA?

***Guo***: I graduated from the Department of Aeronautic Engineering of Northwestern Polytechnical University. I have conducted many years of research into structural engineering in aeronautics and astronautics. As airplane manufacturing started to rely on steel and other metallic materials in the 19th century, engineers began to study the strength and structural safety of these materials. They discovered that alloys such as steel may, after repeated strains, experience fatigue that later leads to cracks. The concept of fatigue used to mainly appear in biology but metals can also get ‘tired’ and experience fatigue. The cause of metal fatigue turned out to be internal non-uniform reaction under external forces. The non-uniformity is necessary as the materials need to be strong and tough, but non-uniformity also causes the accumulation of local plasticity that eventually results in cracks. On a molecular level, cracks originate from broken chemical bonds. Going deeper into atomic and quantum mechanical levels, it is the electrons that bind atoms into molecules and eventually form macroscopic materials. So our research over the past 30 years on the fatigue and cracks of aviation materials put us on a path from mechanics on the macroscopic scale to the microscopic and quantum mechanical scales,

Our research trajectory started with fatigue and cracks on the macroscopic level and extended to microscopic properties such as multi-field coupling at the interface between solids.

—Wanlin Guo

**Figure 1:**
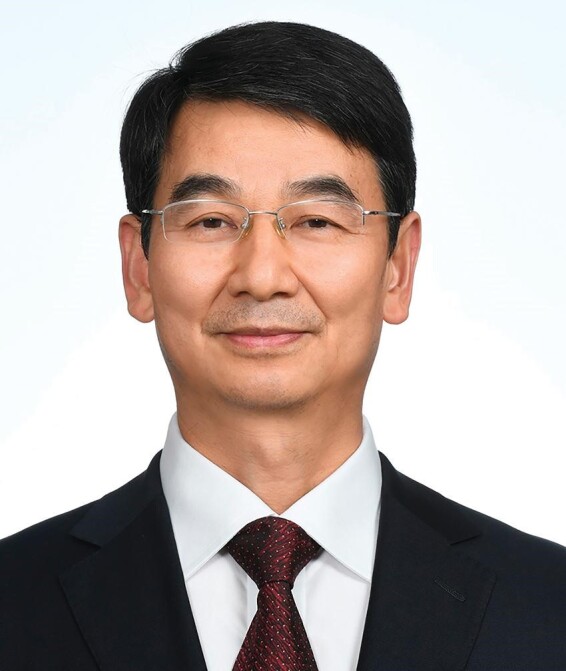
Prof. Wanlin Guo created a new field of science now recognized internationally as ‘hydrovoltaics’ *(Courtesy of Prof. Wanlin Guo).*

and we discovered similar phenomena. For example, carbon nanotubes may elongate up to 20% under an electric field. Further experiments have shown that other external conditions such as force, heat or magnetic fields can change the quantum states of electrons including charge distribution and properties of electron orbits. These changes often initiate at interfaces between heterogeneous regions within materials. Specifically, the toughness of a material depends on the interactions at internal interfaces. In a university of aeronautic research, we naturally focus on interfacial properties such as the interface between a jet engine and air. We investigate how the interactions at this interface affect thrust. Then we expand our experiments to functional materials in water and study how water flow, droplet movement and evaporation interact with electrical charges. Humans’ living environment consists of steps of water circulation such as evaporation, precipitation, river flow and ocean waves. These processes absorb heat and energy from the sun. As we converted this energy directly into electricity through the interaction of water with materials, we gave it the name ‘hydrovoltaics’, similar to photovoltaics. In short, our research trajectory started with fatigue and cracks on the macroscopic level and extended to microscopic properties such as multi-field coupling at the interface between solids. Then we turned our attention to the interface between solid and liquid and discovered hydrovoltaics.

***NSR***: Please introduce the first step in hydrovoltaic experiments that emerged in 2014. How did you discover that a droplet can generate an electrical potential when moving along the surface of graphene?

***Guo***: A student of ours conducted an experiment involving air flow over a graphene surface to generate electricity and then decided to try a similar experiment in liquid. Previous experiments consisting of carbon nanotubes in liquid did generate electricity but our initial experiment of graphene in water flow was not successful. However, we detected a voltage when graphene was taken out of liquid, exposed to the moving interface between air and liquid. In addition, when a droplet moved along the surface of graphene, we detected a voltage that was proportional to the speed of the movement. This discovery received considerable attention around the world when published in 2014 [1].

**Figure 2:**
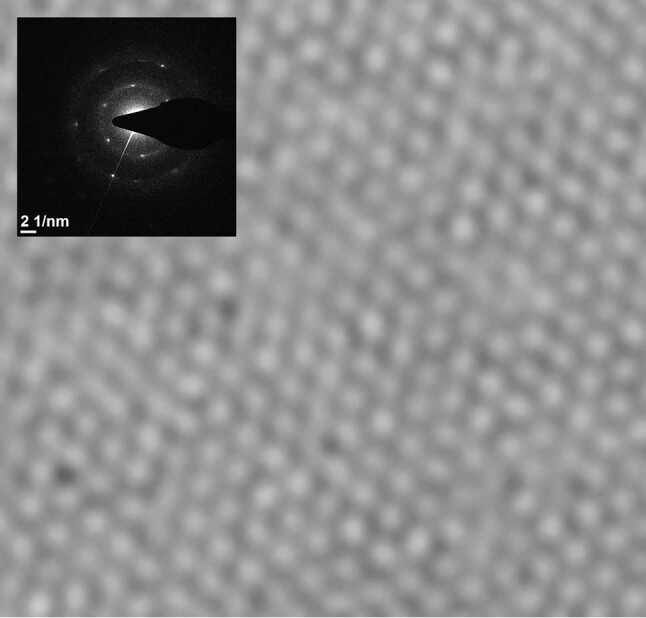
Hexagonal crystal structure of graphene imaged by electron microscopy *(Courtesy of Prof. Guo's research group).*

***NSR***: Your next step in this experiment was to explain the mechanism. So what is the role of graphene in this process?

***Guo***: According to the classic theory of electrokinetics, at the interface of a solid and liquid, negative charges accumulate on the solid surface and they attract positive ions in the liquid to the interface, which is referred to as the adsorption layer. The negative ions in the liquid then form a diffusive layer next to the adsorption layer of the positive ions. In addition, the positive ions also attract more electrons in the solid to the surface and they form a pseudo-capacitor. If a droplet moves along the solid surface, the pseudo-capacitor is continuously charged at the front of the droplet and discharged at the rear of it [2]. This cycle then creates a motion of electrons in the solid and a difference in electrical potential. The positive and negative ions in the liquid form a classic double layer system that needs to include a third layer of electrons in the solid to explain the potential generated between the front and back of the droplet. This three-layer model represents an extension of electrokinetics into energy harvesting.

**Figure 3:**
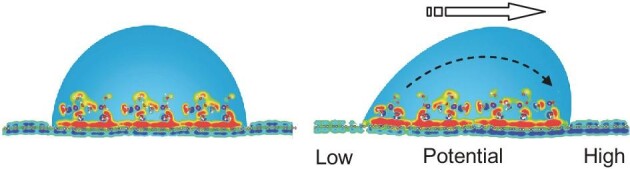
A moving droplet (right) creates a difference of charge potential between its front and rear, in comparison to a static droplet (left). Reproduced from ref. [1] with permission. Copyright 2014, *Springer Nature*.

***NSR***: What factors affect the voltage and power of the electrical output and how can they be maximized?

***Guo***: When we first started experimenting with moving droplets, the output voltage was on the order of millivolts. The output power was around nanowatts. But if we polarized this system with a bias voltage, we further increased the potential generated in graphene. With an optimized circuit, the output was later increased to around 1 volt. Next, we put the droplet on the surface of a dielectric and it formed a capacitor as the droplet spreaded out. Once charge on the capacitor was released to an external circuit, the output was around hundreds of volts. This progress from millivolt to volts to hundreds of volts was achieved in less than 10 years. Recently, our experiment, in which water droplets fell tens of centimeters from a faucet, reached an output voltage of 1200 volts.

***NSR***: The next step in hydrovoltaics is based on evaporation. How does it work and how does it compare to the droplet movement process?

***Guo***: Water on Earth receives 70% of the energy of solar radiation, half of which is consumed in water evaporation. As climate change picks up, the evaporation process increases so there is an enormous amount of energy in the water circulation process that can be harvested to generate electricity. The experiment we designed to demonstrate this process works like this: we put a strip of porous carbon material on a quartz substrate. Then we attached a series of electrodes along this strip and submerged one end of it in water. Naturally, the capillary effect pulls water up along the strip. The variation of voltages we measured across different sections of this strip showed us the characteristics of this phenomenon: the lowest two electrodes both submerged in water showed no voltage and the highest two electrodes both above the capillary range also showed no voltage [3]. Each section within the capillary range showed increasing voltage and the sum from all the sections added up to the total voltage measured between both ends of the strip.

**Figure 4:**
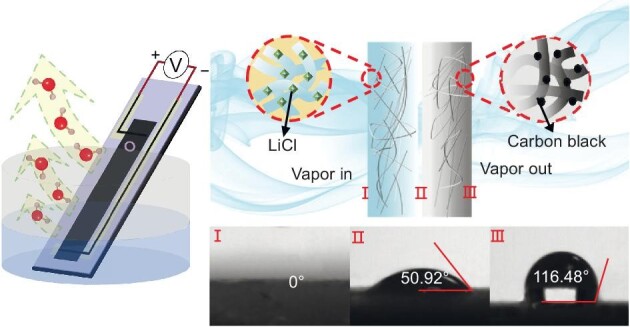
Left: Water evaporation creates a potential gradient along a strip of carbon black. Reproduced from ref. [3] with permission. Copyright 2017, *Springer Nature*. Right: A composite material achieves moisture adsorption with a hygroscopic layer and water evaporation with carbon black. Reproduced from ref. [4] with permission. Copyright 2022, *Springer Nature*.

**Figure 5:**
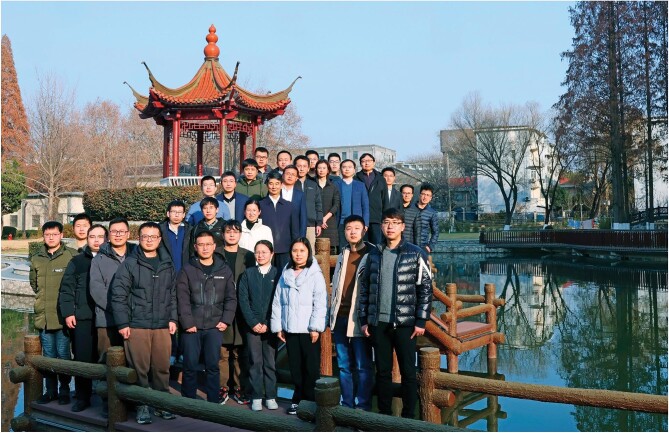
The research group of Prof. Wanlin Guo at NUAA (*Courtesy of Prof. Guo's research group*).

Based on our previous experiments of generating electricity with a moving droplet, we hypothesized that the underlying mechanism of this process is the flow along the strip to sustain water supply in the evaporation process. The next series of experiments showed us the correlation between the voltage we measured and water evaporation. First, when the water was completely sealed in its container, the voltage disappeared within 1000 s; when it was unsealed, the voltage returned. Further, the voltage can be increased with ambient conditions such as wind, increased temperature and decreased humidity. In the natural environment and with the growing influence of climate change, taking advantage of these factors helps us to optimize the evaporation process to harvest maximum energy from the sun and turn it into electricity. If successfully implemented on a large scale, the hydrovoltaic process may alleviate the effects of climate change in addition to providing power.

“As the climate gets warmer, the moisture content of the atmosphere is also higher. To take advantage of this, we proposed a device as a combination of moisture adsorption and water evaporation.

—Wanlin Guo

***NSR***: In a recent development, your group designed a composite material combining moisture adsorption and water evaporation. How much can this improve the efficiency of the hydrovoltaic process?

***Guo***: Water evaporation as a source of electricity may present a challenge because of its inability to sustain itself in the natural environment. For example, in regions without bodies of water such as lakes or rivers, the source of water evaporation must be the atmosphere itself. As the climate gets warmer, the moisture content of the atmosphere is also higher. To take advantage of this, we proposed a device as a combination of moisture adsorption and water evaporation [4]. The function of moisture adsorption was achieved in a hygroscopic layer of cellulon paper impregnated with LiCl, and the evaporation function was achieved in a hydrophobic layer of cellulon paper doped with carbon black. This composite device adsorbs moisture into the layer with LiCl and vaporizes water from the layer with carbon black, resulting in a self-sustainable water cycle. At the same time, the moisture adsorption process releases heat and the evaporation process absorbs heat from the environment, so it also forms a heat cycle. The device can achieve a 10-fold increase in the efficiency of electricity generation, with output potential at the voltage level and output current at the milliamp level.

***NSR***: Can you briefly introduce the latest development in hydrovoltaic materials?

***Guo***: We mentioned graphene used in the droplet experiment and carbon black in the evaporation process. They are both based on carbon-based nanomaterials. Soon we expanded into semiconductors, metal-organic frameworks and biomaterials such as protein membranes. They can all be used as hydrovoltaic materials. The first step of hydrovoltaic research is to discover these processes and what materials allow them to happen. The next step is to identify the ones with higher efficiency and lower costs. For example, the pieces of graphene used in early droplet experiments were transferred onto polymer substrates. The transfer process introduced inconsistency in terms of the quality and number of layers in graphene. Later, we optimized this process by directly synthesizing graphene on a dielectric substrate using chemical vapor deposition and copper acetate as a catalyst [5]. This has greatly improved the graphene quality and made hydrovoltaic materials more suitable for mass production.

Theoretically, we measure our proficiency [in photovoltaic conversion] in terms of electrical power output per unit area, and the current value is about 1 watt per square meter.

—Wanlin Guo

***NSR***: A central issue in photovoltaic conversion is its efficiency. Currently, what is the efficiency of hydrovoltaic conversion?

***Guo***: In photovoltaics, the theoretical maximal efficiency is a bit more than 30%. With a combination of silicon and perovskite technologies, we are approaching that limit. In less than 10 years of developing hydrovoltaics, we have improved the efficiency in the laboratory from about 1/1000 to 10% when we harvest the mechanical energy of water droplets and convert it to electricity. Harvesting the evaporation energy is different because it is a natural process without the need of any external work. The evaporation process is always happening in the water cycle, you may extract as much as your technology allows. Theoretically, we measure our proficiency in terms of electrical power output per unit area, and the current value is about 1 watt per square meter. In terms of efficiency of power conversion in evaporation, if we use all the heat absorbed by liquid water as the denominator, the efficiency is only about one ten-thousandth because most of the heat is consumed by the phase transition of water. So there is much room for improvement. If we can increase that ratio to 1%, we will enter a new age of hydrovoltaic energy for the world.

***NSR***: The topic of your recent research is hydrovoltaic intelligence. Please give us an introduction to that, and how it is related to neuroscience.

***Guo***: We predict that hydrovoltaics will further develop into three categories: hydrovoltaic energy, hydrovoltaic ecology and hydrovoltaic intelligence. Our world has progressed from the mechanical age to the electrical age and then to the information age. Now we are transitioning into the intelligence age. As we examine the source of our intelligence, we see the human brain consisting of 70%–80% water. Our neural network is formed by billions of neurons and each neuron consists of 70%–80% water. How does a system like this receive stimulus, release electrical signals, store memory and eventually form consciousness? This is a major area of future discoveries as we transition from the information age to the intelligence age. What we learn from the human brain may guide the development of artificial intelligence (AI). According to classic neuroscience, electrical charges and neuronal signals in the brain originate from the motions of K, Na and Ca ions in water. However, existing knowledge in ionic dynamics cannot fully explain how the brain generates, stores and processes information. In hydrovoltaics, we investigate the interaction between water and solids from the quantum mechanical level, to the atomic level, to the biomolecular level, and we discover that ionic motions in the brain are modulated or controlled by water. This research direction will combine physics, chemistry and biology. I think future neuroscience will inevitably focus on this area.

Hydrovoltaics takes neuroscience one step further and focuses on how proteins interact with ions and molecules in neurons’ natural environment. This is essential to understanding the transportation of matter and information in the brain.

—Wanlin Guo

***NSR***: You have also investigated the interaction between neurotransmitters and cell membranes. How did you make the transition from artificial 2D materials such as graphene to natural membranes?

***Guo***: In basic sciences we often investigate materials and processes in the natural world. When we look into the activities that happen on cell membranes, the underlying mechanism is quite similar to the hydrovoltaic experiments that generate electricity using artificial materials. These processes all center on the interactions between electrons and ions at the interface between water and solid. We start with the same set of scientific principles from microscopic electron properties to macroscopic properties of electromagnetic fields and heat. Regarding cell membranes, specifically, we know membranes play a central role in the evolution of life, as the regulator of information and materials in and out of cells. These actions are carried out by various proteins on the cell membrane. For example, we recently discovered that neurotransmitters such as dopamine, enkephalin and leucine enkephalin with aromatic side rings were able to freely dip into the membrane of neurons [6]. In contrast, acetylcholine and aspartic acid without an aromatic side ring diffused in the extracellular solution. This functional distinction was achieved by proteins embedded in phospholipid bilayers on the cell membrane.

***NSR***: Structural biologists have studied molecular functions, such as charge transports, on cell membranes. What can hydrovoltaics add to that?

***Guo***: Classic biology first developed into cellular biology with the invention of the microscope. Then molecular biology emerged with the discovery of genes and how genes produce proteins. Cryo-electron microscopy shows us protein structures, so now we are in the era of structural biology. The next step after seeing structure is understanding the correlation between structure and function. Specifically, in neuroscience, we want to know how neurons store and process information. Hydrovoltaics takes neuroscience one step further and focuses on how proteins interact with ions and molecules in neurons’ natural environment. This is essential to understanding the transportation of matter and information in the brain.

***NSR***: When synthesizing artificial materials, what inspiration can we draw from natural materials such as cell membranes?

***Guo***: As we said, an important role of the cell membrane is to regulate energy transport and ionic transport. In hydrogen fuel cells, the crucial function of the proton exchange membrane is quite similar to the cell membrane. In lithium batteries, regulating ionic transport is also key to high performance without damaging internal components. Membrane engineering is now a research focus in many applications of renewable energy.

***NSR***: What do you think are the major milestones in hydrovoltaics and when will they reach practical application?

***Guo***: When we first started hydrovoltaic research with the droplet experiment in 2014 and the evaporation experiment in 2017, we only measured very weak effects. Since 2020, we have managed to improve the kinetic power output by six orders of magnitude and evaporation power output by three orders of magnitude. That is exponential growth within a few years. Along this trend, I am hopeful we may see a breakthrough in 3 to 5 years. However, we must acknowledge that traditional technologies such as hydroelectric power generation and photovoltaic generation took about 100 years to reach commercial application. So it is not possible to predict scientific development before it happens but we are certain new discoveries in physics and chemistry will emerge in this area. In addition, climate change results in more heat and humidity in our environment so humans should invest more in technologies that extract energy from that. Hydrovoltaics may become an enormous source of energy. As for the prospect of hydrovoltaic intelligence, we believe the success of AI depends on what we can learn from natural intelligence.

With machine learning and only a small amount of experimental data, we are now able to predict hundreds of alloys in the composition space of 16 metallic elements.

—Wanlin Guo

***NSR***: You have recently applied machine learning to predicting properties of alloys. Can you give us an introduction to that? How do you see the potential of AI in materials science?

***Guo***: The development of AI started in the middle of the 20th century with the invention of the artificial neural network. With the help of modern computational technologies such as Big Data, AI-based methods such as deep learning or machine learning play an increasingly important role in science. Since the beginning of science in the 15th century, a primary mathematical tool has been differential equations. The problems we aim to solve, from aerodynamics to quantum mechanics, all involve solving differential equations. Under given initial conditions and boundary conditions, we were able to solve these equations with a limited number of parameters. Therefore, our ability to describe the natural world using differential equations has been quite limited. Now with AI we are facing complex materials such as an alloy consisting of as many as 16 metallic elements [7]. The number of parameters required in these problems is beyond traditional mathematical methods but machine learning, combined with Big Data, is able to handle trillions of parameters and simulate complex physical systems in spatial and temporal dimensions. These new research methods will be crucial in developing new functional materials such as new alloys and those used in hydrovoltaic processes. In the past, alloy materials were discovered by trial and error, but it would not be possible to try out all possible combinations of complex formulas of these alloys. With machine learning and only a small amount of experimental data, we are now able to predict hundreds of alloys in the composition space of 16 metallic elements. Furthermore, we are now achieving this with higher precision and a similar amount of computation.

***NSR***: What professional advice do you have for students in mathematics or mechanics?

***Guo***: I'd advise them to build a starting position with a foundational knowledge in classical mechanics, quantum mechanics and mathematics. The best research is conducted with a piece of paper and a pen. In the modern age, you may add a personal computer to help. With those tools and your mind, you want to focus on the cutting edge of a particular subject and make your own contribution to science.

## Notes

He Zhu (朱禾) is a science and news editor at the editorial office of NSR in Beijing, China.
